# The Influence of Whey Protein Heating Parameters on Their Susceptibility to Digestive Enzymes and the Antidiabetic Activity of Hydrolysates

**DOI:** 10.3390/foods11060829

**Published:** 2022-03-14

**Authors:** Kungnang Bunsroem, Witoon Prinyawiwatkul, Siwatt Thaiudom

**Affiliations:** 1 School of Food Technology, Suranaree University of Technology, Nakhon Ratchasima 30000, Thailand; kungnang.ta@rmuti.ac.th; 2 Institute of Agricultural Technology, Suranaree University of Technology, Nakhon Ratchasima 30000, Thailand; 3 School of Nutrition and Food Sciences, Louisiana State University, Baton Rouge, LA 70803-4200, USA; wprinya@lsu.edu; 4 The Louisiana State University Agricultural Center, Louisiana State University, Baton Rouge, LA 70803-4200, USA

**Keywords:** antidiabetic, whey protein, β-lactoglobulin, α-lactalbumin, heat treatment

## Abstract

The inhibition of dipeptidyl peptidase-IV (DPP-IV) and the release of glucagon-like peptide-1 (GLP-1) could normalize blood glucose levels in diabetic patients. This study evaluated the susceptibility of whey proteins to enzyme hydrolysis and the antidiabetic properties of protein hydrolysates from β-lactoglobulin (β-LG) and α-lactalbumin (α-LA) solutions compared with whey protein isolate (WPI) solution treated at different heating temperatures (65, 75, and 85 °C). α-LA hydrolysate provided the lowest degree of hydrolysis (DH). Those heating temperatures did not significantly affect the DH of all protein hydrolysates. α-LA hydrolysate significantly increased GLP-1 levels and DPP-IV inhibitory activity more than β-LG hydrolysate. WPI hydrolysate inhibited DPP-IV activity less than an α-LA hydrolysate, but they were no significant differences for GLP-1 release activity. Heat treatment could affect the antidiabetic properties of all protein hydrolysates. Heating at 75 °C resulted in greater inhibition of the activity of DPP-IV than at 65 and 85 °C. The highest increase in GLP-1 release was also observed by heating at 75 °C. The recently obtained information is useful for the utilization of α-LA, heated at 75 °C for 30 min, in the preparation of antidiabetic food supplements.

## 1. Introduction

Diabetes mellitus (DM) is a metabolic disease that is of worldwide concern. The disease is characterized by hyperglycemia resulting from defects in insulin secretion, insulin action, or both. It severely impairs peoples’ quality of life, attributing to several life-threatening complications, including atherosclerosis, nephropathy, and retinopathy [1,2]. The current therapies for DM mainly include oral antidiabetic drugs and insulin. Some widely used drugs to treat DM patients are, for example, dipeptidyl peptidase-IV (DPP-IV) inhibitors, glucagon-like peptide-1 (GLP-1) analogs, metformin, sulfonylureas, and α-glucosidase inhibitors [3,4]. However, continuous use of these drugs causes insulin resistance and side effects [1]. Thus, the demand for effective, nontoxic, and affordable drugs for DM patients has gained more attention, and natural alternative foods, for instance, milk proteins, have been used for such purposes. 

Whey proteins from milk contain many bioactive peptides. However, commercial whey products are different in their protein content. Whey protein concentrate and whey protein isolate (WPI) contain 65–80% and above 90% of protein on a dry basis, respectively [5]. The major components of WPI are β-lactoglobulin (β-LG) and α-lactalbumin (α-LA) [6]. It was suggested that these bioactive peptides have several health benefits, including antidiabetic [7,8]. WPI in post-simulated gastrointestinal (GI) digestion could inhibit the activity of DPP-IV [9], which is a serine protease expressed in many tissues, such as kidney, liver, lung, and endothelial cells [10]. WPI hydrolysate has been recognized to inhibit DPP-IV activity, which presumably results in an increase in the GLP-1 level, leading to an antihyperglycemic effect [11]. GLP-1 is a hormone that stimulates insulin secretion, which plays a key role in the regulation of blood glucose levels. Insulin deficiency can lead to the development of diabetes symptoms [12]. A different amino acid sequence in WPI has been known as an index of its bioactive capability and potential [13]. α-LA possessed the highest content of short amino acid sequences, which provided the most potential DPP-IV inhibitory activity compared to β-LG, bovine serum albumin (BSA), and lactoferrin, studied in silico. In addition, α-LA in post-simulated GI digestion recognized as α-LA hydrolysate showed a lower level of degree of hydrolysis (DH) than hydrolysates from other whey protein fractions [14,15]. On the other hand, the β-LG hydrolysate was a better source than α-LA hydrolysate as the DPP-IV inhibitor in terms of its half-maximal inhibitory concentration (IC_50_) values [7]. In both previously mentioned methods [7,14], purified β-LG and α-LA were used without consideration of the protein solution after exposure to heat during food processing. Heat treatment is one of the most important processes in food preparation [16]. It could modify the structure of peptides in milk proteins’ powder after hydrolysis, resulting in the inhibition of DPP-IV activity of milk protein hydrolysate in post-simulated GI digestion [17,18]. The heated α-LA showed higher antioxidant activity than the native α-LA during stomach digestion. Nevertheless, the antioxidant activity of β-LG significantly decreased at a temperature above 80 °C [19]. Preheating BSA at the temperature range between 65 and 75 °C could improve the DH values of BSA. However, increasing the temperature above the transition point did not increase the extent of hydrolysis significantly [20]. Importantly, the direct effects of heat treatment of whey protein types on GLP-1 secretion and DPP-IV inhibition after in vitro digestion of these proteins are still unclear and need further investigation. Therefore, this study was aimed at exploring the influence of different types of whey proteins with different heat treatment temperatures on their susceptibility to in vitro enzyme digestion and determining the antidiabetic properties of these protein hydrolysates.

## 2. Materials and Methods

### 2.1. Materials

WPI was purchased from Mullins Whey, Inc. (Mosinee, WI, USA). Both β-LG and α-LA were purchased from Sigma-Aldrich (Saint Louis, MO, USA). The protein content of WPI and α-LA was determined by the Macro–Kjeldahl method [21] using a nitrogen conversion factor of 6.38. WPI and α-LA contained 96.52 ± 0.43 and 96.91 ± 0.87% of protein (dry basis), respectively. The protein content in β-LG was 95%, as determined by Polyacrylamide Electrophoresis (per the manufacturer’s data). Hydrochloric acid and sodium hydroxide were purchased from Carlo Erba Reagents (Val de Reuil, Normandie, France). Dulbecco’s modified eagle medium (DMEM) was bought from GE Healthcare Life Sciences (South Logan, UT, USA). Fetal bovine serum was bought from GE Healthcare Bio-Sciences Austria GmbH (Kremplstrasse, Pasching, Austria). Penicillin/streptomycin solution was bought from Capricorn Scientific GmbH (Auf der Lette, Ebsdorfergrund, Germany). Dimethyl sulfoxide was purchased from Ameresco Inc. (Framingham, MA, USA). GLP-1 Total ELISA kit was obtained from Millipore Corporation (Saint Louis, MO, USA). Other chemicals and enzymes used in this study were of analytical grade and purchased from Sigma-Aldrich (Saint Louis, MO, USA).

### 2.2. Preparation of WPI, β-LG, and α-LA Solution

The powder of WPI, β-LG, and α-LA was dissolved with distilled water to obtain a 50 mg/mL solution. It was heated at 65, 75, and 85 °C for 30 min with constant stirring and then rapidly cooled to 4 °C. The protein solution was stored for no more than 5 days at 4 °C prior to in vitro digestion. The experimental design for this study was a Randomized Complete Block Design (RCBD), with temperatures acting as the treatments and the different types of whey protein solutions assigned as the blocks.

### 2.3. In Vitro Digestion of Whey Proteins

The protein solution in part 2.2 was in vitro digested in a GI simulated system. This was carried out as described by Nongonierma et al. [18]. Briefly, the protein solution was heated at 37 °C for 30 min. The pH of the solution was adjusted to 2.0 using 1 M HCl. The protein solution was hydrolyzed with pepsin (enzyme: substrate (E:S), 2.5% *w*/*w*) for 90 min at 37 °C. Pepsin activity was inactivated by heating the protein solution for 20 min at 90 °C. An aliquot of the peptic hydrolysate was adjusted to pH 7.5 using 1 M NaOH and was subsequently hydrolyzed with pancreatin (E:S, 1% *w*/*w*) for 150 min at 37 °C. The reaction was terminated by thermal treatment (90 °C, 20 min). The protein hydrolysate was stored at 4 °C until further analysis. 

### 2.4. Determination of Degree of Hydrolysis (DH)

The protein hydrolysate in part 2.3 was tested for DH values by 2, 4, 6-trinitrobenzenesufonic acid (TNBS) colorimetry for α-amino nitrogen as described by Yi et al. [22] and Gruppi et al. [23] with a slight modification. Briefly, samples (hydrolyzed and unhydrolyzed control samples after heat inactivation of enzymes) were diluted in 1% (*w*/*v*) sodium dodecyl sulfate to a final protein concentration/protein equivalent of 10 mg/mL and incubated at 50 °C for 60 min. Then, 10 μL of both samples and 10 μL of leucine standards at 0–5 mg/mL were loaded onto a 96-well plate with 80 μL of 0.2 M sodium phosphate buffer, pH 8, followed by 80 μL of 0.025% (*w*/*v*) TNBS. The sample plate was incubated at 45 °C for 30 min in a microplate reader (Thermo Fisher Scientific, Vantaa, Southern Finland, Finland), and the absorbance at 420 nm was monitored every 2 min. The DH values were calculated using the equation as follows:

DH (%) = (*A* − *B*/*T*) × 100.

where *A* is the reactive α-amino nitrogen concentration determined by TNBS colorimetry, *B* is the reactive α-amino nitrogen concentration of the intact protein substrate, and *T* is the total reactive α-amino nitrogen concentration of the intact protein substrate. *B*/*T* values of WPI, β-LG, and α-LA were 0.39/8.29, 0.88/9.24, and 0.92/9.38, respectively [22].

### 2.5. Evaluation of Antidiabetic Properties

#### 2.5.1. Glucagon-like Peptide-1 (GLP-1) Quantification Assay

- Cell culture

Cell lines of the human salivary gland (HSG) were obtained from the laboratory of Dr. Parinya Noisa (School of Biotechnology, Institute of Agricultural Technology, Suranaree University of Technology, Nakhon Ratchasima, Thailand). Cells were grown at 37 °C, with 95% air and 5% CO_2_ in a humidified atmosphere incubator (Thermo Fisher Scientific, Waltham, MA, USA) [24]. Cells were cultured in DMEM containing 4.0 mM L-glutamine and 4.5 g/L glucose supplemented with 10% fetal bovine serum and 1% penicillin/streptomycin [25]. Cells were routinely grown in 75 cm^2^ tissue culture plastic flasks. The DMEM was changed once every 1–2 days [26]. Cells were trypsinized with 0.1% trypsin-EDTA and reseeded. HSG cells were frozen at −80 °C in a freezer (Thermo Fisher Scientific, Marietta, OH, USA) and thawed at 37 °C before a test of cell viability and GLP-1 secretion.

- Cell viability test

The MTT (3-[4,5-dimethylthiazol-2yl]-2,5-diphenyl tetrazolium bromide) colorimetric assay adapted from Arteaga-Cardona et al. [27] was used to determine the cell viability and cytotoxicity. Briefly, the protein hydrolysate was sterilized by filtration through a 0.2 µm PTFE membrane filter (Sigma-Aldrich, Saint Louis, MO, USA). HSG cells were maintained in 96 well culture plates (well growth area of ca. 0.32 cm^2^) at a density of 10^4^ cells/well and incubated in cell culture conditions for 24 h. Then, the DMEM was replaced with 100 µL of fresh DMEM containing different concentrations of the protein hydrolysate (0, 0.078, 0.156, 0.313, 0.625, 1.25, 2.5, 5, and 10 mg/mL). Cells were further incubated for 24 h. Then, the DMEM with protein hydrolysate was removed, and 10 µL MTT solution (5 mg/mL in PBS pH 7.4) was added to each well. After further incubation in the dark for 3 h at 37 °C, the MTT solution was removed, and 100 µL of dimethyl sulfoxide was added to each well. The absorbance was monitored in a microplate reader at a wavelength of 550 nm. The untreated cells were used as the control, and the cell viability was calculated using the following equation:

Cell viability (%) = (Absorbance of sample well/absorbance of control well) × 100.

- GLP-1 secretion test

In order to examine the GLP-1 quantification, HSG cells were maintained at 6 × 10^4^ cells/well in 24 well culture plates (well growth area of ca. 1.86 cm^2^) and incubated in cell culture conditions for 24 h. Then, the DMEM was replaced with 600 µL of fresh DMEM containing the protein hydrolysate at concentration levels which provided the maximum cell viability. Untreated cells were used as the control. After further incubation for 24 h, the supernatants were centrifuged at 664× *g* for 3 min at 25 °C to remove the remaining cells. Total GLP-1 release levels were determined using a GLP-1 total ELISA kit according to the manufacturer’s instruction [28]. 

#### 2.5.2. Dipeptidyl Peptidase-IV (DPP-IV) Inhibition Assay

- Measurement of DPP-IV inhibitory activity

The existence of GLP-1, which was mainly metabolized by DPP-IV, was indirectly determined as the DPP-IV inhibition value [29]. The protein hydrolysate was used at the concentration providing the highest cell viability, as previously mentioned. The protein hydrolysate (25 µL) was mixed with 50 µL of 1 mM reaction substrate (Gly-Pro *p*-nitroanilide hydrochloride) in a 96-well clear microplate. Then, 25 µL of 0.2 units/mL DPP-IV was subsequently added. The microplate was incubated at 37 °C for 30 min, and the absorbance of the *p*-nitroanilide, which was released at 405 nm, was monitored every 5 min in a microplate reader [18]. The results were compared with that of the control (no protein hydrolysate added) [30].

- Measurement of DPP-IV half-maximal inhibitory concentration (IC_50_)

The IC_50_ value of DPP-IV was measured in order to confirm the DPP-IV inhibitory activity of protein hydrolysate. The protein hydrolysate was varied in the range of 5 to 50 mg/mL. Diprotin A (Ile-Pro-Ile) was used as a positive control at the concentration range between 1.56 and 62.50 µg/mL. The DPP-IV IC_50_ value was determined as previously mentioned in the DPP-IV inhibitory activity measurement. The IC_50_ values were determined by plotting the percentage of inhibition and the concentration of protein hydrolysate [18].

### 2.6. Statistical Analysis

All experiments and measurements were performed at least in triplicate. All results were expressed as the mean ± standard deviation. The analysis of covariance with a significance at *p* < 0.05 was used to test the lack of significant differences between the mean of cell viability values at various concentrations of different types of whey protein hydrolysates. A one-way analysis of variance was used to determine the difference between mean values with a significance at *p* < 0.05 for DH percentage, total GLP-1 release, DPP-IV inhibition percentage, and DPP-IV IC_50_ values. A one-way analysis of variance was followed by Duncan’s Multiple Range Test for multiple means comparison. Principal component analysis (PCA) was performed to identify patterns of the experimental data, investigate their similarity and difference, and determine the variation in antidiabetic properties among the different protein hydrolysates. All analyses were determined using the statistical package for the social sciences program (version 17.0, SPSS Inc., Chicago, IL, USA).

## 3. Results

### 3.1. Degree of Hydrolysis of Protein Hydrolysate

**Figure 1:**
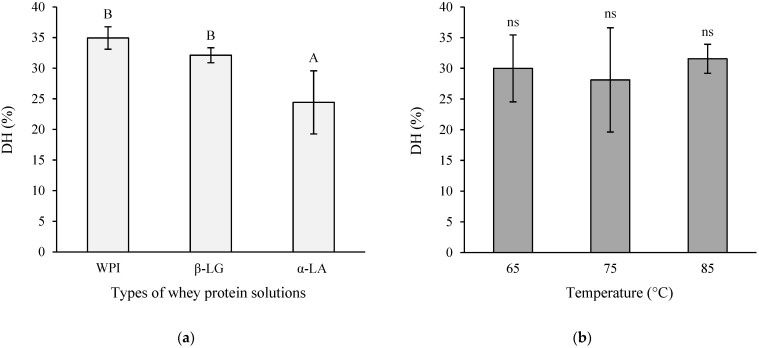
Degree of hydrolysis (DH, %) of protein hydrolysates: (**a**) Comparisons between different types of protein solutions (WPI, β-LG, and α-LA), and (**b**) comparisons between different heat treatment temperatures (65, 75, and 85 °C) for 30 min for all protein solutions. Data are presented as mean ± standard deviation (*n* = 3). The different letters ^A,B^ indicate a significant difference at *p* < 0.05. The letter ^ns^ indicates no significant difference at *p* ≥ 0.05. WPI, β-LG, and α-LA denote whey protein isolate, β-lactoglobulin, and α-lactalbumin, respectively.

The DH values of protein hydrolysates from WPI, β-LG, and α-LA solutions heated at different temperatures (65, 75, and 85 °C for 30 min) were compared. The results are shown in Figure 1. α-LA hydrolysate showed the lowest DH (%) compared to WPI (1.4 times higher) and β-LG hydrolysate (1.3 times higher) (*p* < 0.05). The percentages of DH of WPI and β-LG hydrolysate were not significantly different (Figure 1a). In addition, the heating temperatures did not significantly affect the DH (%, Figure 1b).

### 3.2. Glucagon-like Peptide-1 Release Activity

**Figure 2:**
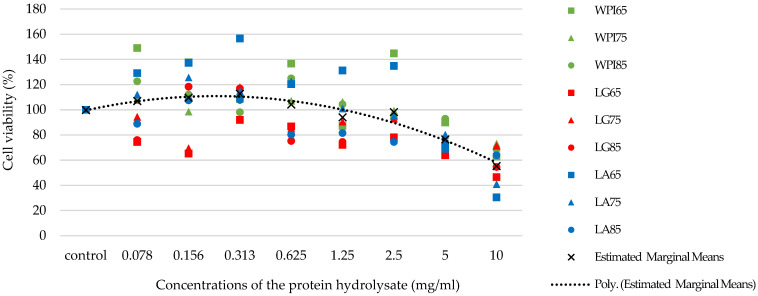
Cell viability (%) of the human salivary gland (HSG) cells after 24 h incubation with DMEM (control) and the protein hydrolysate at concentrations between 0.078 and 10 mg/mL (*n* = 4). WPI, LG, and LA denote whey protein isolate, β-lactoglobulin, and α-lactalbumin solutions, respectively. The number after the abbreviated protein solutions indicates the temperature (°C) of the heat treatment process.

The optimum concentration of the protein hydrolysates from WPI, β-LG, and α-LA solutions at different heat temperatures (65, 75, and 85 °C for 30 min) for GLP-1 quantification was determined using the MTT colorimetric assay. Figure 2 shows that there are two noticeably different zones of cell viability with the protein hydrolysate concentrations beyond 2.5 mg/mL and below. In the present study, significant decreases in cell viability were observed at the high concentrations of all protein hydrolysates (5–10 mg/mL). All protein hydrolysates at the concentration of 0.313 mg/mL were not harmful to HSG cells. Evidently, at this concentration, the cell viability (%) was the highest (92.00–156.64 %, Figure 2). 

The comparison result demonstrated that WPI and α-LA hydrolysates could induce and maintain the cell viability more than a β-LG hydrolysate at the low concentrations (0.078–1.25 mg/mL). Moreover, heating at 65 °C for all protein hydrolysates provided the highest cell viability (86.87–156.64%), compared to those at 75 and 85 °C (75.29–125.71%, Figure 2).

Each protein hydrolysate at three different temperatures providing the highest cell viability was used to determine the GLP-1 release. The results are shown in Table 1. WPI, β-LG, and α-LA hydrolysates significantly increased the release of GLP-1 compared to the control. At all heating temperatures, WPI and α-LA hydrolysates could induce the release of GLP-1 better than β-LG hydrolysate (*p* < 0.05). Furthermore, heat treatment could enhance the release of GLP-1 compared to the control. The release of GLP-1 induced by heating at 75 and 85 °C was not significantly different. However, heat treatment at 65 °C seemed to provide fewer releases of GLP-1 than at 75 and 85 °C (*p* < 0.05).

### 3.3. Dipeptidyl Peptidase-IV Inhibitory Activity

**Figure 3:**
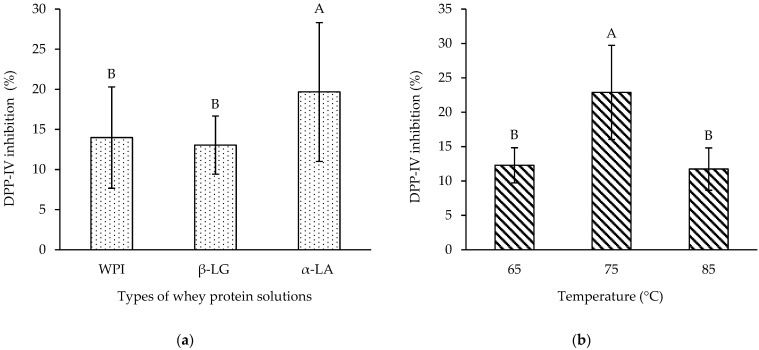
Dipeptidyl peptidase-IV (DPP-IV) inhibition values (%) of protein hydrolysates at a concentration of 0.313 mg/mL: (**a**) Comparisons between different types of protein hydrolysates from WPI, β-LG, and α-LA solutions; (**b**) comparisons between different heat treatment temperatures (65, 75, and 85 °C) for 30 min for all protein hydrolysates. Data are presented as mean ± standard deviation (*n* = 3). The different letters ^A,B^ indicate a significant difference at *p* < 0.05. WPI, β-LG, and α-LA denote whey protein isolate, β-lactoglobulin, and α-lactalbumin, respectively.

The DPP-IV inhibitory activity of the protein hydrolysates from WPI, β-LG, and α-LA solutions at different temperatures of the heat treatment process was determined. The concentration of protein hydrolysate at 0.313 mg/mL, providing the highest cell viability, was used (Figure 2). As shown in Figure 3a, α-LA hydrolysate showed the highest DPP-IV inhibition (%) compared to WPI (1.4 times lower) and β-LG hydrolysate (1.5 times lower) (*p* < 0.05). The percentages of DPP-IV inhibitory activity of WPI and β-LG hydrolysates were not significantly different. Among the three heating temperatures, all protein hydrolysates heat-treated at 75 °C provided the highest DPP-IV inhibitory activity (nearly two times higher) compared to the rest, while there were no significant differences in DPP-IV inhibitory activities at 65 and 85 °C (Figure 3b).

In order to confirm the DPP-IV inhibitory activity of each protein hydrolysate, their DPP-IV IC_50_ values (mg protein/mL) were determined, and the results are shown in Table 2. The DPP-IV IC_50_ value of the α-LA hydrolysate was in the range of 7.16 to 13.80 mg/mL, which was varied with the temperature of the heat treatment process. Regardless of the heating temperature, α-LA hydrolysate could reduce the DPP-IV IC_50_ value better (about two times higher or more) than β-LG and WPI hydrolysate (*p* < 0.05). For each protein hydrolysate, there were no significant differences in DPP-IV IC_50_ values at 65 and 85 °C (*p* ≥ 0.05), but these values were significantly (*p* < 0.05) lower than those at 75 °C. Diprotin A was used as a reference for DPP-IV inhibitory activity, and its DPP-IV IC_50_ value was 0.0185 ± 0.0010 mg/mL in this current study.

### 3.4. Principal Component Analysis

**Figure 4:**
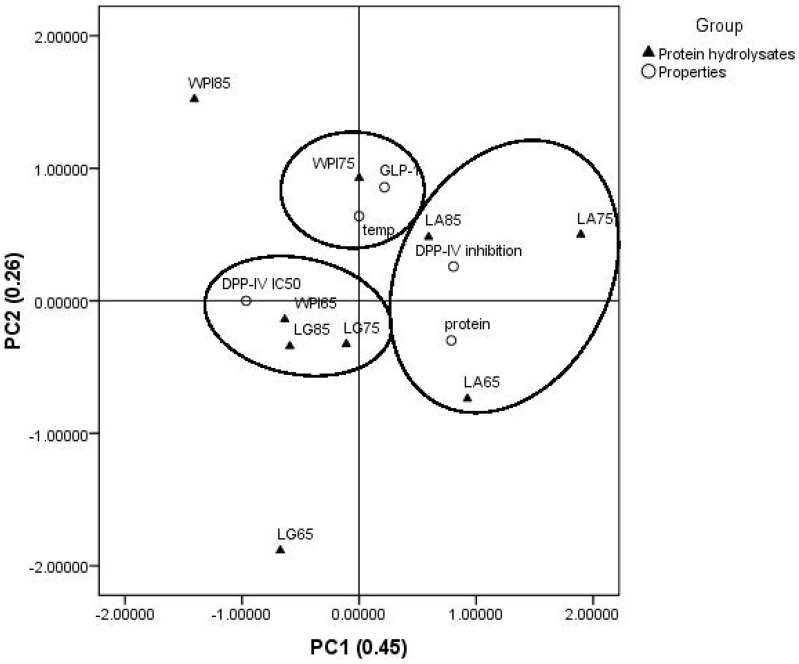
Bi-plot for the protein hydrolysates and their properties from principal component analysis (PCA). WPI, LG, and LA denote whey protein isolate, β-lactoglobulin, and α-lactalbumin solutions, respectively. The number after the abbreviated protein solutions indicate the temperature (°C) of the heat treatment process.

In order to investigate the relationship between the protein hydrolysates from different types of protein solutions with different heat treatment temperatures and their antidiabetic properties, PCA was performed. In Figure 4, the PCA biplot of the first and second principal components (PC1 and PC2) for the antidiabetic properties of the nine protein hydrolysates is presented. The sum of PC1 and PC2 explained 0.71 (71%) of the total variance, which was higher than the acceptable variance explained (0.60) in factor analysis for a construct [31]. As shown in the PC1, a group of the α-LA hydrolysates with different heat treatment temperatures was attributed to an influential type of protein solution with its best DPP-IV inhibition. In addition, the effect of 75 °C heating temperature on high GLP-1 release was grouped by the PC2. This PCA plot suggests that protein hydrolysates from three protein solutions (WPI solution with the heating temperature at 65 °C as well as β-LG solutions with the heating temperatures at 75 and 85 °C) are correlated by the DPP-IV IC_50_ values. This is supported by the data in Table 2, which shows that the DPP-IV IC_50_ value of protein hydrolysates from WPI solution with the heating temperature at 65 °C, and β-LG solutions with heating temperatures at 75 and 85 °C were 24.19 ± 0.85, 25.35 ± 1.10, and 26.57 ± 0.70 mg/mL, respectively.

## 4. Discussion

This study explored the impact of whey proteins heating parameters, which were types of protein solutions and heat treatment temperatures, on the susceptibility to digestive enzymes and the antidiabetic activity of hydrolysates. The α-LA hydrolysate showed the lowest DH value, compared with WPI and β-LG hydrolysates. This result was consistent with the findings of Corrochano et al. [15] and Lagace [32]. The higher the DH value, the higher the susceptibility of the protein hydrolysate to digestive enzymes. This might be due to the effect of different protein structures. A β-sheet structure, found in β-LG, which possesses more simple structure and more accessible sites for the digestive enzyme than an α-helix form of α-LA, could be easier hydrolyzed by the enzymes [33,34]. Moreover, β-LG is the main component found more than α-LA in WPI. Thus, the DH value of WPI hydrolysate was not significantly different from that of β-LG hydrolysate.

The result of the effect of the temperature process showed that the DH values from WPI, α-LA, and β-LG hydrolysates were not significantly different. This might be because when those proteins were hydrolyzed with the same type of enzymes and hydrolysis time, finally, the particle size of protein hydrolysates should provide the same particle size distribution, resulting in the non-significantly different DH values. However, Arrutia et al. [20] revealed that the higher DH values of BSA hydrolysate were attributed to the preheated temperature between 65 and 75 °C but not at 85 and 95 °C.

HSG cells were used as an in vitro model of GLP-1 release. This cell line has been widely used to elucidate the mechanism of GLP-1 release [35,36,37]. However, food digestion is known to be cytotoxic to cells, which depends on the type, concentration, processing, quantity of food, etc. [38,39]. The viability of HSG cells was negatively correlated with concentrations of all protein hydrolysates between 2.5 and 10 mg/mL. In addition, at those concentrations, the cell viability dramatically decreased because such a high concentration of those hydrolysates could be toxic to cells. This was relevant to the generally used-optimum concentration range of the tested compounds, which was between 1.0 × 10^−4^ and 0.5 mg/mL [27,32,38]. At the concentrations of protein hydrolysates below or equal to 2.5 mg/mL, WPI and the α-LA hydrolysate heated at 65 °C for 30 min, known as a low-temperature long time (LTLT) pasteurization [40], induced better cell viability compared to the control baseline. However, when heating was at 75 and 85 °C, each protein hydrolysate did not clearly induce cell viability. Corrochano et al. [41] explained that direct exposure of cell lines to whey protein hydrolysate increased intracellular antioxidants such as glutathione, which was recognized as a diet for the well-being of cells. This is in line with the previous findings that α-LA did not display the cell membrane-damaging activity in an aqueous solution at a neutral pH. However, there was no evidence of the effect of α-LA hydrolysate on cell viability [42].

An increase in GLP-1 released from HSG cells was found in the protein hydrolysates from the protein solution samples heated at 65, 75, and 85 °C. This means that all heated protein hydrolysates used in this study could inhibit the activity of DPP-IV. In addition to the effect of temperature that increased GLP-1 release, the insulinotropic effect of whey protein hydrolysates was believed to delay the inactivation of GLP-1 release induced by DPP-IV [7]. However, the types of whey proteins greatly influenced their antidiabetic properties. The peptide profiles undoubtedly inserted a role for these bioactivities after the simulated GI digestion [9]. We observed that α-LA hydrolysate significantly increased GLP-1 release level more than β-LG hydrolysate (Table 1). This was because α-LA hydrolysate exhibited a higher potential on the in vitro DPP-IV inhibitory activity than β-LG hydrolysate. The shorter amino acid residues of whey peptides contributed to their best DPP-IV inhibitory activity. The best DPP-IV inhibitor was peptides with a length of 3–6 amino acids [43,44]. However, practically, tri-peptides IPI (diprotin A) and VPL (diprotin B) have always been used as the precursor and standard of the DPP-IV inhibition [45]. Regarding the DH values of these protein hydrolysates, the DH of α-LA hydrolysate was the lowest compared to the others. This implies that α-LA hydrolysate was the most resistant to the action of these studied digestive enzymes. The lower DH value seemed to correlate with a lower amino acid content but not with a higher amount of short-chain dipeptides, which could inhibit DPP-IV activity [46]. Thus, α-LA hydrolysate showed less DH but high DPP-IV inhibitory activity. Even though WPI hydrolysate could inhibit DPP-IV activity less than α-LA hydrolysate, both were not significantly different in the induced GLP-1 release (Table 1). This might be due to the modified structure of GLP-1 by glycine in WPI hydrolysate, which may be resistant to enzyme degradation by DPP-IV [47,48].

The structural changes in whey protein were recognized as a consequence of heat treatment [49]. Hydrophilic interactions of proteins (hydrogen bonds, Van der Waals interactions, electrostatic interactions between charged groups, and specific binding) were weakened, while their hydrophobic interactions were strengthened by heat treatment in the temperature range of 60 to 80 °C [50]. At natural pH (4.5–6.0) values, β-LG was denatured by temperature between 75 and 80 °C [16]. The thermal denaturation temperature of α-LA was, on average, at 63.7 °C [51]. The denaturation ratio of whey proteins increased approximately by 20, 45, and 98% at the heating temperatures of 65, 75, and 85 °C for 30 min, respectively [52]. However, three heating temperatures did not significantly affect the susceptibility of protein hydrolysates to digestive enzymes. In contrast, heating at 65 °C gave less GLP-1 release than heating at 75 and 85 °C, which was not significantly different in GLP-1 levels (Table 1). In addition, the effect of the heat treatment process on the DPP-IV inhibitory activity of the protein hydrolysates was clearly evidenced in this study (Table 2 and Figure 3). Protein hydrolysates from heating the protein solution at 75 °C resulted in a significantly higher DPP-IV inhibition percentage and lower IC_50_ value than at 65 and 85 °C.

The data of all results evaluated by PCA revealed that there were three groups based on the relationship between antidiabetic properties and protein hydrolysates. The first group showed that α-LA hydrolysate could be the most effective substrate to inhibit DPP-IV (Figure 3a and Figure 4), while the second group revealed that heating at 75 °C could induce the highest level of GLP-1 release (Table 1 and Figure 4). The last one was the group of protein solutions with no clear correlation with their DPP-IV IC_50_ value (Table 2 and Figure 4).

## 5. Conclusions

This study revealed that α-LA provided the least DH of hydrolysate compared to β-LG and WPI. The α-LA hydrolysate exhibited antidiabetic properties better than WPI and β-LG hydrolysates. However, all protein hydrolysates were harmless to HSG cells at a specific concentration, that is, 0.313 mg/mL. Evidently, α-LA hydrolysate possesses a high potential for being the enhancer for GLP-1 release and DPP-IV inhibitory activity when a thermal process is applied. Heating at 75 °C for 30 min gave the best results of GLP-1 release and DPP-IV inhibition while heating at 65 and 85 °C for 30 min provided both lower activities of GLP-1 release and DPP-IV inhibition. From the PCA analysis, process temperature could affect GLP-1 release while the types of whey protein hydrolysate influenced DPP-IV inhibitory activity. Conclusively, α-LA solution heated at 75 °C for 30 min can potentially be used as a potential antidiabetic substance in the form of a food supplement for diabetic patients in the future. However, the optimal concentration of α-LA solution heated at 75 °C for 30 min should be further investigated for such purpose. In addition, the effect of preheating temperature and enzyme inactivation temperature on antidiabetic activity of protein hydrolysate as well as the physicochemical properties, e.g., size distribution, structure, and bioactive peptides and amino acid sequences of such protein hydrolysates should be further investigated.

## Figures and Tables

**Table 1:** Glucagon-like peptide-1 (GLP-1) release values (pM) of protein hydrolysates from whey protein isolate (WPI), β-lactoglobulin (β-LG), and α-lactalbumin (α-LA) solutions with different heat treatment temperatures. Untreated cells were used as the control.

Temperature (°C)	Types of Whey Protein Hydrolysates			
	WPI	β-LG	α-LA	Control
65	12.37 ± 0.06 ^Ba^	11.98 ± 0.08 ^Bb^	12.34 ± 0.02 ^Ba^	
75	12.43 ± 0.08 ^Aa^	12.20 ± 0.07 ^Ab^	12.41 ± 0.05 ^Aa^	
85	12.51 ± 0.06 ^Aa^	12.08 ± 0.02 ^Ab^	12.37 ± 0.07 ^Aa^	
Control				11.83 ± 0.06 ^Cc^

Data are presented as mean ± standard deviation (*n* = 6). The different letters ^A,B,C^ in the same column indicate a significant difference at *p* < 0.05. The different letters ^a,b,c^ in the same row indicate a significant difference at *p* < 0.05.

**Table 2:** The Dipeptidyl peptidase-IV half-maximal inhibitory concentration (DPP-IV IC_50_) values (mg/mL) of protein hydrolysates from whey protein isolate (WPI), β-lactoglobulin (β-LG), and α-lactalbumin (α-LA) solutions with different heat treatment temperatures.

Temperature (°C)	Types of Whey Protein Solutions		
	WPI	β-LG	α-LA
65	24.19 ± 0.85 ^Bb^	30.81 ± 0.89 ^Bb^	12.31 ± 0.52 ^Ba^
75	20.41 ± 1.64 ^Ab^	25.35 ± 1.10 ^Ab^	7.16 ± 0.82 ^Aa^
85	37.40 ± 0.36 ^Bb^	26.57 ± 0.70 ^Bb^	13.80 ± 0.87 ^Ba^

Data are presented as mean ± standard deviation (*n* = 3). The different letters ^A,B^ in the same column indicate a significant difference at *p* < 0.05. The different letters ^a,b^ in the same row indicate a significant difference at *p* < 0.05.

## Data Availability

The datasets generated for this study are available on request to the corresponding author.
